# Does food safety perception matter to farmers’ happiness？ evidence from China

**DOI:** 10.1016/j.heliyon.2023.e23868

**Published:** 2023-12-19

**Authors:** Zhong-kun Zhu, Chen-xin Leng

**Affiliations:** a School of Humanities and Social Sciences, Beijing Institute of Technology, Beijing, China; b Academy of Agricultural Planning and Engineering, Ministry of Agriculture and Rural Affairs(MARA), Beijing, China

**Keywords:** Conditional mixed process method, Food safety perception, Happiness, Recursive bivariate ordered probit model, Rural China

## Abstract

Although the determinants of individuals' happiness have been widely examined in the literature, little is known regarding whether and how food safety perception affects farmers' happiness. To fill this research gap, this paper examines the impact of food safety perception on happiness among Chinese farmers, utilizing open-access data collected through the Chinese Social Survey project in 2013, 2017 and 2021. This study focuses on Chinese farmers as the research subject, attempting to analyze the “happiness code” from the perspective of food safety, which supplements the literature on happiness and provides reference for protecting the rights of low-income groups and promoting food safety strategies in developing countries. To address sample selection bias, this paper employs the recursive bivariate ordered probit (RBOP) model and conditional mixed process (CMP) method. The results reveal that the perception of food safety exerts a positive and statistically significant impact on farmers' happiness in China. In addition, food safety perception is more important among middle-aged and elderly farmers and among those with higher education. Thus, the policy makers should continue to make up for the shortcomings of rural food safety work and extend regulatory measures to rural areas. They also need to take efforts to strengthen food safety promotion, enhance farmers' safety awareness, and safeguard farmers’ “safety on the tongue”.

## 1 Introduction

Hunger breeds discontentment, safety is the supremacy for food, according to an old Chinese saying. Food safety is related to people's health and the future of the Chinese nation (Xi, 2014). The report of the 20th National Congress of the Communist Party of China emphasizes the necessity to strengthen food safety supervision. The first document of the Central Government in 2023 further elaborated on the need to strengthen the supervision of food safety and agricultural product quality safety, and improve the traceability management system. However, from melamine to gutter oil to clenbuterol, public discussions of problems in connections with food safety have continued, seriously affecting people's physical and mental health, even causing a decline in social credibility [1,2]. The Research Group of the Chinese People's Livelihood Survey of the Development Research Center of the State Council (2018) notes that food safety is China's foremost socio-environmental concern. Strengthening food safety governance continues to be called for by urban and rural residents, which indicates that food safety is of substantial practical significance to the people and the entire society.

In recent years, China has made remarkable achievements in economic construction, with a continuous increase in per capita income. But meanwhile, China has fallen into the dilemma of “stagnation of happiness”. Easterlin's study on China showed that income growth and residents' happiness were not synchronized in transition countries. Between 1990 and 2009, China's economic growth rate was about 8 %, while the happiness status of Chinese residents during the same period showed an inverted U-shaped pattern. The happiness level of rural residents in 2009 was even lower than that in 1990 [3]. The results of the World Values Survey also indicated that the happiness level of Chinese didn't increase synchronously with economic growth [4]. China has also been adjusting its economic policies, focusing on ensuring and improving people's livelihoods. To this end, the academic community has been investigating how to crack the “happiness code” to enhance people's happiness. From economic factors to social factors, from micro factors to macro factors, scholars have performed numerous in-depth studies on happiness. However, food safety, a major concern of daily living, has received little attention from scholars. Furthermore, it is worth noting that although China's per capita GDP has reached 12,000 US dollars, the wealth gap is still significant, with a Gini coefficient of 0.474 in 2022, with the urban-rural gap being the most significant.^2^ Many rural residents have difficulty ensuring food safety, and a safe food supply is crucial for them. Therefore, the following three research questions were considered. What role does food safety play in the process of achieving a happy life for the general public? How deeply does food safety affect farmers' happiness, and what are the characteristics of the influence?

To answer these questions, this paper focuses on the impact of food safety perception on farmers' happiness. This paper not only provides a more substantial basis for decision-making to promote food safety governance measures but also has strong practical significance for building a harmonious society and enhancing people's sense of progress, security and happiness.

## 2 Literature review

### 2.1 Theory as the ground of this study

Happiness is the ultimate goal of human beings, which is an overall evaluation of personal quality of life and subjective feedback on their inner state [5,6]. Happiness is also a permanent topic among scholars. More than 2000 years ago, Aristotle proposed that happiness was the ultimate end or object of human life and the epitome of all good [7].

Early economics, especially classical economists, believed that economic growth and wealth increase were the only factors that increased people's happiness. They established the foundation for studying economic activities on people's subjective psychological feelings, and regarded enhancing people's well-being and happiness as the ultimate goal of economic activities. Adam Smith, believed that economics should aim for the happiness of human beings as its ultimate goal. Utilitarian master [8] proposed on the basis of cardinal utility theory that the focus of economics was on how to maximize people's happiness. Since the 1920s [9], has established welfare economics, and welfare economists have replaced “happiness” with “welfare” to study economic activities. However, after the 1930s, as ordinal utility theory gradually replaced cardinal utility theory, the concept of “happiness” gradually faded away in economics, and utility gradually lost its connotation of happiness.

In the 1950s, psychologists began to focus on the study of happiness. More recently, positive psychology, a branch of psychology that aims to improve individual happiness, has emerged [10,11]. [12] pioneered the concepts of decision utility and experiential utility, drawing on positive psychology. They pointed out that in current economic decision-making theory, utility refers to decision-making utility, while actual feelings such as happiness and pain are experiential utility. From this the development of positive psychology reintroduces the exploration of the essence and sources of happiness into the framework of scientific analysis.

In 1974 [13], directly focused on happiness in economic research and creatively proposed the Easterlin Paradox. He observed that within a country, people with higher incomes find it easier to obtain happiness, but at the national level, the average happiness of a country does not improve with the growth of per-capita GDP, thus creating a clear paradox. This paradox has created a precedent for the systematic study of happiness and a new direction in economics: happiness economics.

Since the emergence of the Easterlin Paradox, scholars have tried to crack the happiness code from different perspectives [14]. From absolute income to relative income to income inequality, income has been an important focus of scholarly attention [5,[15], [16], [17]]. In addition, there have been a variety of studies on the relationship between happiness and socio-demographic factors, such as age, gender, education, marital status, health status, housing status and religious beliefs [6,[18], [19], [20]]. Macroeconomic factors of substantial significance to happiness include unemployment, inflation and environmental pollution [[21], [22], [23]]. In recent years, institutional factors, such as democratic development and government quality, have been linked to happiness [24,25]. In addition, the economic impact of happiness has received more and more attention [26,27]. A number of studies discusses the relationship between food and happiness [[28], [29], [30]], but few papers focus on the impact of food safety on individuals’ happiness.

### 2.2 Food safety

Food safety affects the entire nation and has long been a concern of all sectors of society. As time has passed, the concept of food safety has been enhanced, and the perception of food safety worldwide has been evolving. Overall, the focus of food safety perception has undergone a transition from food quantity safety to food quality safety. In 1974, the Food and Agriculture Organization (FAO) first defined food safety as the amount of food required to maintain people's basic needs. In 1996, the World Health Organization (WHO) regarded food safety as a guarantee of consumer health when food was produced and consumed according to original purpose. In 2003, FAO/WHO further noted that food safety referred to all hazards that may make food harmful to health. In addition, foreign scholars have put forward their own definitions of food safety. According to Ref. [31]; food safety refers to the opposite of food risk in a narrow sense, that is, the possibility of eating a food without contracting a disease. In a broad sense, food safety also includes the nutritional quality of food, and more extensive attention paid to unusual food characteristics [32]. stress that food safety means that the health of consumers may be harmed by food as a result of chemical residues, antibiotics or food additives in food. Over time, food safety issues have also become a focus for domestic scholars [33]. propose that since China's reform and opening up, the trajectory of change in China's food safety concerns can be described as “Food quantity safety→Food quantity and hygiene→Food quality safety→Food quality and nutrition safety”. With the continuous increase in the consumption level of urban and rural residents, the desire of urban and rural consumers has shifted from the pursuit of food quantity to the pursuit of food quality, safety and health.

As the understanding of food safety has increased, relevant research in the field of food safety has been constantly emerging. Scholars have offered many insights on the meaning, causes and supervision of food safety issues. In terms of food safety supervision, scholars have engaged in numerous discussions (Martinez et al., 2007; [2,34]. The root cause of food safety problems is market failure caused by information asymmetry. Information asymmetry tempts enterprises to imitate low-quality competitors, which can trigger an industrial food safety crisis (Rouviere & Soubeyran, 2008). The “short-sighted cognitive bias” of enterprises amplifies the “false impulse” to produce fraudulent and inferior products [2]. From the perspective of government regulation [34], emphasize that the policy burden results in the weakening of regulation, which can lead to an absence of government regulation. Most previous studies believe that China's food safety governance should adopt the basic strategy of “combination and coordination of compatible short-term containment measures and long-term strategies” to overcome a food safety crisis [2]. Short-term measures focus on the establishment of a company blacklist system, with regulations regarding repeated offenses, including heavy penalties and supervision mechanisms for offending companies. Long-term measures stress the improvement of relevant laws and information-disclosure mechanisms, the enhancement of the food regulatory system and government-internal governance, and the establishment of third-party governance structures, such as media and industry associations (Zhou & Wang, 2013). Zhou & Wang (2013) further propose that in addition to the construction of a supervision system, the food safety basic education system should be improved and a food safety integrity system established.

Despite the wealth of research in the field of food safety, studies on the relationship between food safety perception and happiness remain scarce, and even quantitative assessments of the effects of food safety on individual or family welfare are rare [35]. analyze the potential relationship between food safety and farmers' production technical behavior choices based on 331 household questionnaire data from Guangdong Province. From the perspective of producer welfare, Wen & Han (2018) examine the “yield premium” effect of enterprise participation in food safety regulation. In terms of the relationship between food safety and happiness, Li & Wang (2015) use food safety as a control variable to investigate the influencing factors of happiness, which is not targeted. Tang (2017) uses 2016 survey data of urban residents in Jiangsu and Shandong provinces of China to investigate the impact of food safety perception on happiness. Tang's study represents a useful examination of the relationship between food safety and happiness. In contrast with this paper, Tang's study mainly focuses on urban residents of Jiangsu Province and Shandong Province, while failing to address the endogeneity problem in detail. Thus, the robustness of Tang's conclusions must be reconsidered.

In summary, there has been a lack of attention and insufficient explanation regarding the important questions of how food safety affects people's happiness, particularly with respect to whether and to what extent food safety affects farmers' happiness. Therefore, the study attempts to analyze the “happiness password” from the perspective of food safety, which is an important supplement to literature on happiness.

### 2.3 Hypothesis

As mentioned earlier, happiness is the ultimate goal of people's lives, and it can be said that happiness comes from a sense of inner satisfaction and security. And safe food supply is the most basic human demand, and in China, there are still some farmers whose food safety has not been fully met.

It is not difficult to infer that food safety will bring joy, satisfaction, and security to farmers, thereby enabling them to achieve happiness. Therefore, the paper proposes a research hypothesis:

Hypothesis: Food safety perception can help improve farmers’ happiness.

## 3 Methods

### 3.1 Design

This paper utilizes the Ordered Probit model to investigate the impact of food safety perception on farmers’ happiness, using the data collected from the China Social Survey (CSS). To address sample selection bias, the paper employ propensity score matching (PSM) method, the recursive bivariate ordered probit (RBOP) model and the conditional mixed process (CMP) method. Next, this paper will introduce the Population and Sample, Procedure in details.

### 3.2 Population and sample

This study uses open-access data provided by the China Social Survey (CSS),^3^ hosted by the Institute of Sociology of the Chinese Academy of Social Sciences.^4^ The survey was conducted nationwide based on PPS (Probability Proportionate to Size) sampling, covering various aspects such as labor and employment, family and social life, and social attitudes, which was launched in 2006 and the latest released data is for 2021.

Only three years of data, 2013, 2017, and 2021, involve food safety perception. Therefore, this article utilisizes the data from these three years. It is worth noting that CSS2017 did not directly set happiness variable, but instead set a highly correlated “life satisfaction” variable. Although CSS2021 sets variables for happiness and life satisfaction, the forms of the two variables are different from CSS2013, and the two variables are distributed in the A and B volumes of CSS2021, which limited the sample size. By contrast, the CSS2013 data is the most suitable for analyzing the relationship between food safety and farmers’ happiness, so this paper uses it as the main data for empirical analysis. CSS2017 and CSS2021 are mainly used for robustness check of empirical models.

The CSS2013 data includes 596 villages from 149 counties in 30 provinces nationwide, with 10,206 valid questionnaires collected. The CSS2017 data includes 576 villages from 151 counties, with 10,091 valid questionnaires collected. The CSS2021 data includes 592 villages from 152 counties, with 10,136 valid questionnaires collected. The paper selected samples living in rural areas. In CSS2013, 6271 valid samples were obtained. Similarly, In CSS2017, 6727 valid samples were obtained. In CSS2021, 2946 valid samples were obtained.

### 3.3 Procesure

#### 3.3.1 Modeling the food safety perception effect on farmers’ happiness

Happiness is a discrete ranking data. The paper use the orderd probit (Oprobit) model as the benchmark model. The paper assume happiness variable to be a function of a food safety perception variable (FSP) and a vector of explanatory variables (X):

(1) $${Happiness}^{*}=\beta FSP+\gamma X+\varepsilon \;\text{with}\;Happiness=\left\{ \begin{array}{c} 1,if\;{Happiness}^{*}\leq C_{1}\;\\ 2,if\;{C_{1}<Happiness}^{*}\leq C_{2}\\ \cdots \\ 6,if\;C_{5}\leq {Happiness}^{*}\; \end{array}\right.$$

where ${Happiness}^{*}$ is an unobserved latent variable representing farmers' happiness, represented by an observed categorical variable Happiness. This latter is determined by the unknown cutoffs $C_{1}$, $C_{2}$, …, $C_{5}$, which satisfy the condition that $C_{1}$ <$C_{2}<\cdots <C_{5}$. FSP indicates food safety perception, and X is a vector of explanatory variables. β and γ are parameters to be estimated. ε is a random error term.

#### 3.3.2 Propensity score matching method

Given that farmers' food safety perception is likely to be a self-selection process determined by individual characteristics (e.g., farmers’ age, education level, employment status and income level). Thus, this paper use PSM method to construct the counterfactual framework to correct the sample selection bias. Propensity score is proposed by Ref. [36]; which is defined as the conditional probability that an individual is affected by some explanatory variables after controlling the observed variables. It should be noted that PSM method requires treatment variable is binary. Therefore, the paper redefine the variable of food safety perception, and define “very unsafe and unsafe” as “unsafe”, and assigns it to 0. Similarly, it defines “safe and very safe” as “safe” and assigns it to 1. Within the sample range, there are 3790 farmers who think food safe, accounting for 54 %, and 3229 farmers who think food unsafe, accounting for 46 %.

As shown in equation (2), P(X) is the propensity score, X is a vector of observed variables. The dummy variable FSP={0,1}, known as the treatment variable, indicates whether the farmer reports food safe or not. $\frac{\exp\;\left(\gamma X\right)}{1+\exp\;\left(\gamma X\right)}$ is the cumulative distribution function of the logical distribution, γ is the parameter to be evaluated, which is used to obtain the propensity score P(X).

(2) $$P\left(X\right)=\mathrm{Pr}\left[FSP=1|X\right]=\frac{\exp\;\left(\gamma X\right)}{1+\exp\;\left(\gamma X\right)}$$

As shown in equation (3), the paper compare the average difference in farmers' happiness between the treatment group and the matched control group based on the matched samples, and obtain the causal effect between food safety perception and farmers’ happiness, that is, the average treatment effect on treated (ATT).

(3) $$\tau_{ATT}^{psm}=E\left[\left({Happiness}_{1}-{Happiness}_{0}\right)|FSP=1\right]=E\left\{E\left[\left({Happiness}_{1}-{Happiness}_{0}\right)|FSP=1\right],P\left(X\right)\right\}$$

It is also worth noting that the PSM method has limitations [37]. If the first stage model is set up incorrectly or if the observable variables are chosen improperly or too few, estimation bias can easily result. For this reason [37], notes that caution is required when estimating the use of the propensity matching method.

Based on PSM method and referring to Ref. [38]; this paper uses inverse probability weighting to modify the model. Inverse probability weighting (IPW) is similar to the basic principle of PSM method. However, IPW does not directly use propensity scores to estimate but, rather, assigns higher weight to individuals with lower propensity scores and lower weight to individuals with higher propensity scores. This approach contributes to the closer distribution of the covariates of the treatment group and the control group and more robust ATT results. In addition, scholars have optimized IPW, typically represented by inverse probability weighting-regression adjustment (IPWRA) [39]. The largest advantage of this method is that the estimation results have double robustness; that is, as long as one of the first-stage equations and the result equations are correct, the ATT estimation results are consistent [38,39]. The ATT of the IPW method and IPWRA method can be shown in equations (4), (5).

(4) $$\tau_{ATT}^{IPW}=E\left[\left({Happiness}_{1i}-{Happiness}_{0i}\right)|{FSP}_{i}=1\right]=\frac{1}{N}\sum_{i=1}^{N}{Happiness}_{i}\frac{{FSP}_{i}-P\left(X_{i}\right)}{1-P\left(X_{i}\right)}$$

(5) $$\tau_{ATT}^{IPWRA}=E\left[\left({Happiness}_{1i}-{Happiness}_{0i}\right)|{FSP}_{i}=1\right]=\frac{1}{N}\sum_{i=1}^{N}{Happiness}_{i}\left\{\left[\frac{P\left(X_{i}\right)}{1+P\left(X_{i}\right)}|{FSP}_{i}=1\right]-\left[\frac{P\left(X_{i}\right)}{1+P\left(X_{i}\right)}|{FSP}_{i}=0\right]\right\}$$

#### 3.3.3 Recursive bivariate ordered probit (RBOP) model

The PSM, IPW and IPWRA methods can correct the sample selection bias caused by observed variables, but it does not account for unobserved factors when serving to address the issue of selection bias. The RBOP model proposed by Ref. [40]; in contrast, can sufficiently address selection bias by taking into account both the observed and unobserved factors and then estimating the impact of food safety perception on farmers’ happiness. We also adopt a conditional mixed process (CMP) approach for its robustness checks.^5^

The RBOP model jointly estimates Equation (1), which models the food safety perception and farmers’ happiness, and Equation (6), a latent model whose observable components enable indirect expression of the utility difference.

(6) $${FSP}^{*}=\varphi +\eta S+\mu \;with\;FSP=\left\{ \begin{array}{c} 1,if\;{FSP}^{*}\leq D_{1}\;\\ 2,if\;D_{1}<{FSP}^{*}\leq D_{2}\\ \;\ldots \\ 4,if\;D_{3}\leq {FSP}^{*} \end{array}\right.$$

where ${FSP}^{*}$ is a latent variable to measure food safety perception which cannot be directly observed, but ${FSP}^{*}$ has a certain quantitative relationship with FSP. This latter is determined by the unknown cutoffs $D_{1}$, $D_{2}$, $D_{3}$, which satisfy the condition that $D_{1}$ <$D_{2}<D_{3}$. FSP indicates food safety perception, and S is a vector of explanatory variables, φ and η are parameters to be estimated, and μ is a random error term.

As shown in equation (7), The RBOP model estimates Equations (1), (6) simultaneously using a full information maximum likelihood (FIML) estimator [40] and under the assumption that the random errors (μ,ε) follow a bivariate normal distribution across individuals with a zero mean and unit variance.

(7) $$\left(\begin{array}{c} \mu \\ \varepsilon \end{array}\right)∼N\left\{\left(\begin{array}{c} 0\\ 0 \end{array}\right),\left[\begin{array}{c} 1\;\rho_{\mu \varepsilon }\\ \rho_{\mu \varepsilon }\;1 \end{array}\right]\right\}$$

When $\rho_{\mu \varepsilon }$ is significantly different from 0, it indicates that the model has an endogenous problem, that is, the estimation result of RBOP model is better than that of Oprobit model. On the contrary, if $\rho_{\mu \varepsilon }$ is not significantly different from 0, then the estimation results can be obtained by reference to the Oprobit model.

Although the RBOP model can be identified on the non-linearity of the system, for a better identification, the food safety perception equation should include a variable that is absent from the farmers' happiness equation, one that serves as an instrumental variable (IV) affecting food safety perception but having no direct impact on farmers’ happiness. This paper employ the frequency of farmers reading newspapers as an IV. Considering that RBOP model can not directly test the validity and effectiveness of IV, the paper use the two-stage least square estimation method (2SLS) to test it.

### 3.4 Data analytic

The dependent variable is farmers’ happiness, which was addressed in the survey questionnaire in the following manner. Survey participants were asked to respond to the statement “Overall, I am a happy person” by choosing from the following response options: very unhappy, unhappy, not very happy, relatively happy, happy, very happy and hard to say. This paper delete the response “hard to say” and assign the other six items scores of 1, 2, 3, 4, 5 and 6, respectively.

As shown in Table 1, in the CSS2013 data, the number of farmers who responded with “relatively happy” is the largest, which is 2666 accounting for 37.26 %. The number of farmers who responded with “happy” ranked the second, which is 2126 accounting for 29.71 %. In contrast, the number of farmers who responded “very unhappy” was the least (144), with the lowest proportion (2.46 %).

**Table 1:** Distribution of farmers’ happiness.

Happiness	Very unhappy	Unhappy	Not very happy	Relatively happy	Happy	Very happy
Frequency	144	515	919	2346	1882	465
Proportion (%)	2.30	8.21	14.65	37.41	30.01	7.42

***Note:*** The contents in the table are calculated based on CSS data.

**Fig. 1:**
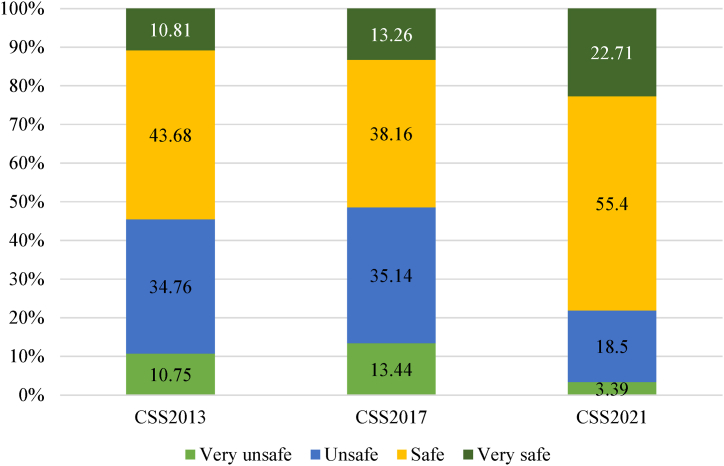
Farmers' food safety perception in data from different years. *Note:* Data sourced from China Social Survey.

The independent variable is food safety perception. Respondents were asked, “How do you feel about the safety of food in the current society?” and were requested to respond by choosing from very unsafe, unsafe, safe, very safe and hard to say. This paper delete the response “hard to say” and assign the remaining four items scores of 1, 2, 3 and 4, respectively. The higher that the value is, the safer the respondents believe food is, that is, the higher the level of food safety perception. Fig. 1 demonstrates farmers' food safety perception in data from different years of 2013, 2017 and 2021. It shows that in the CSS2013 data, the mean value of food safety is 2.547, while in the CSS2017 and CSS2021 data, the mean values are 2.505 and 2.974, respectively. This means that over time, farmers’ food safety perception has improved.

**Fig. 2:**
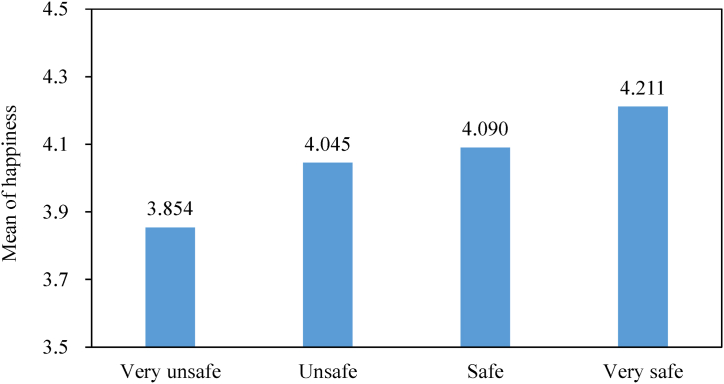
Relationship between food safety perception and farmers' happiness *Note*: Data sourced from China Social Survey.

As shown in Fig. 2, in the CSS2013 data, there is a positive correlation between farmers’ food safety perception and their happiness. Specifically, the average happiness level of the farmers who replied “unsafe” was only 3.854 compared with 4.211 (i.e., 9.26 % higher) for the farmers who replied “very unsafe”. Of course, the logical relationship between food safety perception and happiness requires rigorous empirical analysis.

Based on the CSS2013, this paper also controls for a number of variables that may affect farmers’ happiness (Table 2), including age, age squared, education, gender, marital status, political status, employment status, income, Internet use, economic status, and social trust. In addition, given regional heterogeneity, this paper also controls for regional dummy variables.

**Table 2:** Descriptive Statistics.

Variables	Definitions		Mean (S.D.)^1^	
		CSS2013	CSS2017	CSS2021
Happiness	Very unhappy = 1, very happy = 6	4.059 (1.137)	–	–
Happiness	Very unhappy = 1, very happy = 4	–	–	3.379 (0.687)
Life satisfaction	Very dissatisfied = 1, Very satisfied = 10	–	6.630 (2.234)	–
Food safety perception	Very unsafe = 1, very safe = 4	2.535 (0.827)	2.505 (0.882)	2.974 (0.740)
Age	Age of respondent	45.914 (13.648)	46.429 (14.089)	46.612 (14.242)
Age square	Age square/100	22.944 (12.306)	23.541 (12.623)	23.755 (12.817)
Education	Years of education	7.097 (3.822)	7.667 (4.082)	8.528 (4.079)
Gender	Male = 1, female = 0	0.438 (0.496)	0.454 (0.498)	0.437 (0.496)
Marriage status	Married = 1, unmarried = 0	0.917 (0.276)	0.894 (0.308)	0.888 (0.316)
Political identity	Party member = 1, mass = 0	0.101 (0.302)	0.127 (0.334)	0.141 (0.348)
Employment status	Employment = 1, unemployment = 0	0.779 (0.415)	0.671 (0.470)	0.567 (0.496)
Income level	farmers' income (Logarithmic form)	9.033 (2.229)	–	9.251 (1.163)
Internet use	Use = 1, no use = 0	0.221 (0.415)	0.333 (0.471)	0.638 (0.481)
Economic status	Down = 1, up = 5	2.293 (0.914)	1.929 (0.886)	2.272 (0.936)
Social trust	Very disagree = 1, very agree = 4	3.018 (0.563)	–	–
Social trust	Very disagree = 1, very agree = 10	–	6.163 (1.964)	6.930 (2.191)
Frequency of farmers reading newspapers	Never = 0, nearly every day = 5	0.762 (1.456)	–	–
Mental state	Never = 1, always = 5	2.130 (0.968)	–	–
Life satisfaction	Very dissatisfied = 1, very satisfied = 6	3.753 (1.169)	–	–

***Notes:***^1^ S.D. refers to standard deviation. The contents in the table are calculated based on CSS data.

## 4 Results and discussion

### 4.1 Benchmark regression

In Table 3, columns (1)–(3) list the OLS model estimates, whereas columns (4)–(6) list the Oprobit model estimates. Overall, the model runs well, with the F value and the Wald test values both passing the significance test at the statistical level of 1 %. Additionally, there is no significant change in the direction and significance level of the independent variable between the columns, indicating that the model estimates are robust.

**Table 3:** Benchmark regression: OLS model and Oprobit model.

Variables	OLS model			Oprobit model		
	(1)	(2)	(3)	(4)	(5)	(6)
Food safety perception	0.099***	0.081***	0.084***	0.013***	0.011***	0.012***
	(0.018)	(0.019)	(0.019)	(0.002)	(0.002)	(0.002)
Age		−0.021**	−0.022***		−0.003**	−0.003***
		(0.008)	(0.008)		(0.001)	(0.001)
Age square		0.028***	0.028***		0.004***	0.004***
		(0.009)	(0.009)		(0.001)	(0.001)
Education		0.010**	0.007*		0.001**	0.001*
		(0.004)	(0.005)		(0.001)	(0.001)
Gender		−0.046	−0.025		−0.006	−0.003
		(0.030)	(0.029)		(0.004)	(0.004)
Marriage status		0.295***	0.260***		0.037***	0.033***
		(0.070)	(0.069)		(0.009)	(0.009)
Political Identity		0.160***	0.142***		0.022***	0.020***
		(0.051)	(0.051)		(0.007)	(0.007)
Employment status		0.071**	0.076**		0.010**	0.010**
		(0.035)	(0.035)		(0.005)	(0.005)
Income level		0.014**	0.025***		0.002**	0.003***
		(0.007)	(0.007)		(0.001)	(0.001)
Internet use		0.127***	0.111***		0.017***	0.014***
		(0.042)	(0.042)		(0.006)	(0.006)
Economic status		0.297***	0.294***		0.038***	0.037***
		(0.016)	(0.016)		(0.002)	(0.002)
Social trust		0.319***	0.296***		0.043***	0.040***
		(0.030)	(0.030)		(0.004)	(0.004)
Constant	3.811***	1.967***	2.540***			
	(0.047)	(0.208)	(0.274)			
Provinces	No	No	Yes	No	No	Yes
F value/Wald test	30.832***	55.467***	22.618***	33.779***	586.965***	788.060***
R^2^/Pseudo R^2^	0.005	0.108	0.141	0.002	0.038	0.050
Observations	6271	6271	6271	6271	6271	6271

***Notes:*** ***p < 0.01, **p < 0.05, *p < 0.1. Standard errors are presented in parentheses. The contents in the table are calculated based on CSS data.

Through comparison, the paper can observe that whether according to the OLS model (which regards happiness as a continuous variable) or the Oprobit model (which considers the intrinsic ranking of happiness) the estimation results reveal that food safety perception is significantly positively correlated with farmers’ happiness, even after adding control and regional dummy variables. In column (6), the probability of farmers responding “very happy” increases by 1.2 %, and the average level of farmers responding “very happy” (7.46 %) increases by 16.09 % for each unit of improvement in food safety perception.

These outcomes reveal that the significance of food safety perception to farmers' happiness is not only reflected on the statistical level but also in the real economy. To enhance farmers’ happiness and break free from the “stagnation of happiness” state, the policy makers should consider taking food safety as the starting point and promoting food safety regulatory policies.

In terms of the control variables, most of the variables also significantly affect farmers' happiness, and the estimation results are basically consistent with the literature. Next, this paper briefly analyzes the estimation results based on column (6). The influence of age on the happiness of farmers displays a typical U-shaped distribution. The lowest point of U-shaped distribution, the “turning point of happiness”, is approximately 38 years of age. A possible explanation of this finding is that in youth, people have less stress, lighter burdens, and a stronger sense of happiness. As they grow older, pressure at home and work increases, and happiness decreases to the lowest point. Subsequently, happiness increases with age. The farmer's mental state gradually becomes peaceful, and the sense of happiness improves. Education level, income level, economic status and social trust are significantly positively related to farmers' happiness. In addition, whether a farmer is married, a party member, employed or an Internet user has a significant positive impact on the farmer's happiness. However, considering that certain control variables may face potential endogeneity problems, one must be careful regarding the above estimates.

### 4.2 Correction of selection bias

Using the PSM method, a balance test is required to ensure that after matching there is no significant systematic difference between the treatment group and control group of samples except for differences among food safety perception variables [41]. noted that there should be no systematic differences in the distribution of explanatory variables between the treatment group and the control group after matching. Therefore, pseudo R^2^ should decrease significantly, and the LR test of the explanatory variables should be rejected. In addition [42], observed that after matching, the standardization bias of explanatory variables decreases dramatically. Generally, the normalization coefficient of the explanatory variables after matching should be less than 20 %, and a coefficient higher than 20 % means that the matching process has failed.

As shown in Table A1, after the matching was completed, a balance test was performed. Prior to the matching, pseudo R^2^ was 0.056, and the LR test outcome was 499.02, which is significant at a statistical level of 1 %. The average and median of the standardized errors were 22.1 % and 15.4 %, respectively. After the application of different matching methods, pseudo R^2^ decreases to 0.002 and below. The results of LR test are not statistically significant, and the average and median of the standardization errors are not higher than 2.3 %. These results indicate that the PSM significantly weakens the systematic differences in explanatory variables and that the matching process is successful.

**Fig. 3:**
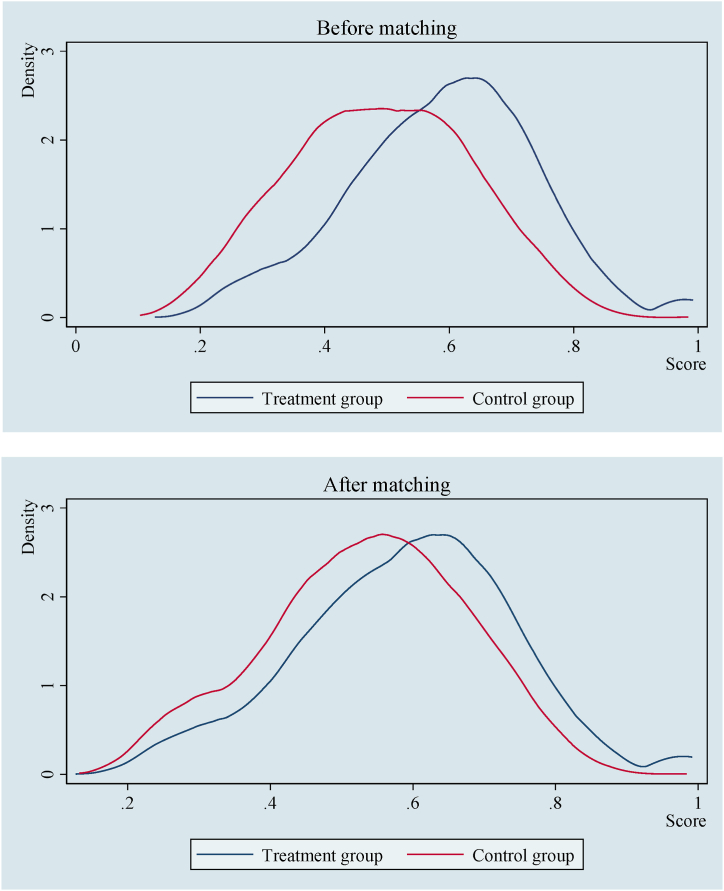
Distribution of propensity scores. *Note:* The contents in the figure are calculated based on CSS data.

To ensure matching quality, the paper also establish the probability distribution of the propensity scores of treatment group and control group before and after matching. As shown in Fig. 3, the difference in the probability distribution of the two groups of samples prior to matching is extremely significant, and the overlap interval of the two groups of samples is narrow. After matching, the difference between the two groups of samples is significantly smaller, with a considerable range of overlap interval. In this way, the proportion of effective samples lost is very low, and the matching quality is satisfactory. This result further confirms that the PSM method can correct the sample selection bias and more accurately assess the causal effect between food safety perception and farmers’ happiness.

Finally, this paper measures the ATT of two sets of samples after matching (Table 4), although different matching methods produce different quantitative results. Qualitatively, the results of the different matching methods are consistent. That is, after observing systematic differences between samples, food safety perception and farmers’ happiness are significantly positively related. It is worth noting that in the process of radius matching, the matching accuracy constantly improves and reduces the caliper range. Although there is a certain “loss” of samples, the ATT estimation results remain robust.

**Table 4:** PSM method estimates.

Matching method	Treatment group	Control group	ATT
Neighbor matching (k = 1)	4.129	4.009	0.120 (0.044)***
Neighbor matching (k = 4)	4.129	4.011	0.118 (0.036)***
Local linear matching	4.129	3.994	0.135 (0.044)***
Kernel matching	4.129	4.005	0.124 (0.033)***
Radius matching (radius = 0.01)	4.127	4.002	0.126 (0.033)***
Radius matching (radius = 0.005)	4.126	4.001	0.125 (0.033)***
Radius matching (radius = 0.001)	4.124	4.027	0.097 (0.034)***

***Notes:*** ***p < 0.01. Standard errors are presented in parentheses. The contents in the table are calculated based on CSS data.

In addition, as shown in Table A2, this paper uses IPW method and IPWRA method to calculate ATT. The results reveal that ATT as obtained by IPW method and IPWRA method differs from that obtained by PSM method in numerical level but is highly consistent in significance and direction. This outcome also confirms the robustness of the empirical results.

### 4.3 Endogeneity discussion

Based on the preceding discussion of the potential endogeneity problems of the model, this paper uses the RBOP model and the CMP method to analyze and reduce the potential impact of endogeneity problems. As shown in Table 5, generally, the estimation results obtained by the two methods are very close, which reveals the consistency and robustness of the estimation results. More specifically, in the first stage, the frequency of farmers reading newspapers is significantly positively correlated with food safety perception, indicating that the instrumental variable satisfies the correlation condition. Further, ρ_με_ is significant at the statistical level of 1 %, indicating that the model has endogeneity problems and that the RBOP model is superior to the ordinary model. Based on the results of the second stage, food safety perception is significantly positively related to farmers’ happiness. This outcome is consistent with the preceding estimation results, indicating that these results are robust and reliable.

**Table 5:** Estimates based on the RBOP model and CMP method.

Variables	RBOP model		CMP method	
	Stage 1	Stage 2	Stage 1	Stage 2
Frequency of farmers reading newspapers	−0.074 (0.011)***		−0.078 (0.010)***	
Food safety perception		0.625 (0.117)***		0.501 (0.156)***
$\rho_{\mu \varepsilon }$		0.682 (0.145)***		0.540 (0.169)***
Control variables	Yes	Yes	Yes	Yes
Provinces	Yes	Yes	Yes	Yes
Log likelihood	−16,058.442		−16,154.735	
Wald test	1206.67***		992.37***	
Observations	6271		6271	

***Notes:*** ***p < 0.01. Standard errors are presented in parentheses. The contents in the table are calculated based on CSS data.

### 4.4 Robustness checks

#### 4.4.1 Add control variables

Both the independent variable and the dependent variables are subjective variables. Hu (2019) notes that the largest challenge in explaining one subjective variable with another is that these subjective variables may have a common basis for potential psychological traits, which may result in falsely related problems. He further emphasizes that if psychological traits are independent, that is, two subjective variables are generated by different psychological traits, they will not be constrained by the same psychological trait and cause confusion and bias.

Although food safety perception and happiness are both subjective evaluations of respondents, there are significant differences in their psychological basis. The perception of food safety often “tells the truth” with stronger relevance, mostly regarding the respondents’ actual feelings on food safety issues or their attention to policy information and personal observation. In contrast, happiness is a quite comprehensive inner feeling. Additionally, the psychological state of the respondents is likely to affect food safety perception and happiness. Therefore, this paper attempts to add psychological state variables in the robustness test. In the CSS2013 questionnaire, respondents are asked, “Overall, how often do you feel worried (scared)?” They answered by choosing from the options never, rare, sometimes, often, always, which are assigned scores of 1, 2, 3, 4 and 5, respectively, as proxy variables of the mental state. This paper further add the fairness perception variable to the test. Respondents were asked, “What do you think of the overall social equity situation?” The response options were very unfair, not fair, fair, very fair, which are assigned scores of 1, 2, 3 and 4, respectively.

As shown in Table A3, mental state and fairness perception have a significant impact on happiness. When the variables of mental state and fairness perception are added, the regression coefficient of food safety perception variable decreases but still has a significant positive impact on happiness.

#### 4.4.2 Omitted variable test

In addition to the previously mentioned addition of mental state and fairness perception variables for robustness testing, this paper also refers to the method proposed by Ref. [43] to test the unobservable variables in the regression [43]. demonstrated that when there are unobservable omitted variables in the model the estimate $\beta^{*}$ = $\beta^{*}\left(R_{\max,\delta }\right)$ can be used to obtain a consistent estimate of the true coefficients.

$R_{\max}$ refers to the maximum goodness of fit of the regression equation when the unobservable omitted variables can be observed, and δ refers to the selection balance between observable and unobservable variables. Specifically, there are two methods for testing as follows. In method (1), the paper assume that $R_{\max}$ is a certain value (generally, $R_{\max}$ is 1.3 times the goodness of fit of the current regression). On this basis, the paper calculate the value of δ that makes β=0. If δ is greater than 1, the estimation result passes the test. Method (2) is similar to method (1). When the paper assume that $R_{\max}$ is a certain value, if $\beta^{*}$ = $\beta^{*}\left(R_{\max,\delta }\right)$ falls within the 95 % confidence interval of the estimated parameters, the estimated results pass the test. As shown in Table A4, the results of this paper are robust and reliable according to the test of omitted variables.

#### 4.4.3 Regression analysis based on CSS2017 and CSS2021

In addition to the CSS2013 data, the CSS2017 and CSS2021 data released by the Institute of Sociology of Chinese Academy of Social Sciences also involves variables related to food safety and happiness. Therefore, the paper use the CSS2017 and the CSS2021 data to test the robustness of the regression results. It is worth noting that, slightly different from the CSS2013, the CSS2017 data does not directly relate to respondents' happiness, but examines respondents’ life satisfaction. Specifically, the questionnaire asks respondents: Overall, how satisfied are you with your life. Respondents choose an integer from 1 to 10, with 1 being very dissatisfied and 10 being very satisfied. Using the life satisfaction index to measure happiness is also a method commonly used in existing literature [44]. Although the CSS2021 sets variables for happiness and life satisfaction, these two variables are set differently from CSS2013, and due to variable limitations (happiness and life satisfaction are random a and b volumes, with sample size halved), CSS2013 is still used as the benchmark regression analysis.

As shown in Table A5, the estimation results of OLS regression and Oprobit model based on the CSS2017 and CSS2021 show that farmers’ food safety perception exerts a positive and significant impact on their happiness or life satifacation. Even after adding other explanatory variables and regional dummy variables, the finding of a positive relationship between food safety perception and happiness or life satifacation confirms the results presented in the above part of Table 3.

In addition, as shown in Table A2, this paper uses IPW method and IPWRA method to calculate ATT (Table A3 in the Appendix). The results reveal that the ATT obtained by IPW method and IPWRA method differs from that obtained by PSM method in numerical level but is highly consistent in significance and direction. This finding further confirms the robustness of the empirical results.

### 4.5 Further analysis

Although the paper have concluded that food safety perception has a significant positive effect on farmers’ happiness, the paper has only obtained the average effect of the entire sample, without considering the heterogeneity between groups. Therefore, this section of the paper focuses on educational and intergenerational differences between subgroups to develop a more detailed discussion.

In terms of educational differences, this paper divides farmers into two groups according to education level: those who have received junior high school education or below (lower education level group) and those who have received senior high school education or above (higher education level group). Within the sample range, there are 1082 farmers with higher education (15.25 %) and 6012 farmers with lower education (84.75 %). As shown in Table 6 generally, the impact of food safety perception on the happiness of farmers with different education levels is significant at the statistical level of 1 %. Specifically, the impact of food safety perception on the happiness of farmers with a higher education level is more obvious, which indicates that food safety is more important for farmers with such education levels.

**Table 6:** Further analysis——educational differences and intergenerational differences.

Variables	Educational differences		Intergenerational differences		
	Low level of education	Higher level of education	Youth	Middle	Elderly
Food safety perception	0.017***	0.021***	0.016***	0.015***	0.035***
	(0.003)	(0.007)	(0.004)	(0.003)	(0.008)
Control variables	Yes	Yes	Yes	Yes	Yes
Provinces	Yes	Yes	Yes	Yes	Yes
Wald test	473.935	–	182.894	297.750	–
Pseudo R^2^	0.033	0.039	0.032	0.033	0.047
Observations	5498	1013	2212	3164	1135

***Notes:*** ***p < 0.01. Standard errors are presented in parentheses. The contents in the table are calculated based on CSS data.

A possible explanation for this result is that highly educated farmers know more about food safety issues, and because they have a better understanding of these issues, they pay more attention to them. Therefore, food safety perception plays a more important role with respect to their happiness.

In terms of intergenerational differences, this paper divides the sample into three groups according to age: young, middle-aged and elderly. Here, young people are farmers under 40 years old, middle-aged farmers are 40–60 years old, and elderly farmers are over 60 years old. Within the sample range, there are 2504 young farmers (34.43 %), 3488 middle-aged farmers (47.96 %), and 1281 elderly farmers (17.61 %). As shown in Table 6, food safety has a significant positive impact on the happiness of farmers of different ages. From the regression coefficient perspective, food safety has the most significant impact on the happiness of elderly farmers, whereas the impact on the well-being of young and middle-aged farmers is approximately the same.

One reason why elderly farmers are more sensitive to food safety may be that with increasing age the health of the elderly is not as good as that of young and middle-aged individuals. Life experience enables the elderly to better understand the truth that illness “comes from the mouth”. Therefore, the elderly are significantly more sensitive to food safety issues than young and middle-aged individuals.

In conclusion, there is educational and intergenerational heterogeneity in the impact of farmers’ food safety perception on happiness. Therefore, in the process of filling the gaps in rural food safety, it is necessary to mobilize highly educated farmers to become food safety regulators and strengthen the promotion of food safety among the elderly in rural areas.

## Data availability statement

The data that support the findings of this study are available from the corresponding author, Chenxin Leng (lengchenxin@163.com), upon reasonable request.

## 5 Conclusions and insights

This paper systematically examines the impact of food safety perception on farmers' happiness, using the data collected from the China Social Survey in 2013. The empirical results show that farmers' perception of food safety is significantly positively related to their level of happiness, and this effect continues after adding control variables and considering regional heterogeneity. Besides, considering that farmers' perception of food safety is the result of self-selection, the paper use PSM, IPW, and IPWRA methods to correct sample selection bias. This paper control for potential endogeneity problems using the RBOP model and the CMP method. Given the problems of omitted variables, mental state variables and fairness perception variables are added to the model, and the [43] method is used to test the impact of the omitted variables on the empirical results. The final conclusion remains stable. Furthermore, the results of the expansibility analysis further indicate that educational and intergenerational differences affect the impact of food safety perception on farmers’ happiness. Food safety perception is even more important in the more highly educated, middle-aged and older farmers.

The core contribution of the study lies in its use of authoritative empirical evidence to confirm the positive effect of food safety perception on farmers' happiness and to suggest an important means for farmers to find their “road to happiness”. At present and for a period in the future, continuing to strengthen food safety and sparing no effort to advance food safety strategies should be an important policy focus for achieving farmers’ happiness. Specifically, the paper must compensate for food safety shortcomings in the countryside, strengthen the establishment of professional food safety teams at the grass-roots level, and gradually improve food safety supervision in countryside. At the same time, it is of substantial significance to disseminate food safety publicity, improve the perception of food safety risks of farmers (particularly the middle-aged and elderly groups), advocate the idea that everyone is responsible for food safety supervision, and mobilize farmers (particularly those with higher education levels) to become food-safety “supervisors”. In the long run, the policy makers should establish a food-safety supervision system based on strict laws, strict supervision, severe punishment and a serious accountability mechanism to truly realize the “safety on the tip of the tongue” of farmers and guarantee the “road to happiness” for farmers.

Finally, it should be pointed out that there are still some shortcomings in this paper. Firstly, farmers' understanding of happiness varies from person to person, and their perception of food safety also differs. However, due to the fact that CSS databases are not tracking data, the paper cannot capture the dynamic changes in farmers' food safety perception and happiness, making it difficult to more accurately identify the causal effects between the two variables. Secondly, China is promoting food safety regulations, and different regulatory models and intensities may lead to the changes in farmers' food safety perception. However, due to data limitations, the paper cannot examine the impact of regulations on farmers' perception of food safety. Thus, the future research should conduct tracking research on farmers, and attempt to develop a questionnaire to examine the impact of different regulatory models and intensities on farmers’ food safety perception and happiness.

## CRediT authorship contribution statement

**Zhong-kun Zhu:** Writing - review & editing, Resources, Project administration, Methodology, Investigation, Funding acquisition, Data curation. **Chen-xin Leng:** Writing - review & editing, Writing - original draft, Visualization, Validation, Supervision, Software, Resources, Formal analysis, Conceptualization.

## Declaration of competing interest

The authors declare that they have no known competing financial interests or personal relationships that could have appeared to influence the work reported in this paper.

## Figures and Tables

**Table A1:** Balance test of PSM method

Matching method	Pseudo R^2^	LR test	P>χ^2^	Mean Bias (%)	Med Bias (%)
Before matching	0.056	499.02	0.000	22.1	15.4
Neighbor matching (k = 1)	0.002	15.64	0.155	2.3	1.3
Neighbor matching (k = 4)	0.001	6.97	0.801	1.9	1.9
Local linear matching	0.002	14.82	0.139	2.3	1.2
Kernel matching	0.000	2.12	0.995	0.9	1.0
Radius matching (radius = 0.01)	0.000	3.23	0.975	1.1	1.3
Radius matching (radius = 0.005)	0.000	3.66	0.961	1.1	1.0
Radius matching (radius = 0.001)	0.000	3.04	0.981	1.0	1.1

***Note:*** The contents in the table are calculated based on CSS data.

**Table A2:** IPW method and IPWRA method estimates

Estimation method	ATT	*t* value
IPW	0.125 (0.030)***	4.25
IPWRA	0.129 (0.030)***	4.36

***Notes:*** ***p < 0.01. Standard errors are presented in parentheses. The contents in the table are calculated based on CSS data.

**Table A3:** Robustness checks——add control variables

Variables	Add mental state variable		Add fairness perception variable	
	OLS model	Oprobit model	OLS model	Oprobit model
Food safety perception	0.127***	0.017***	0.082***	0.010***
	(0.020)	(0.003)	(0.021)	(0.003)
Mental state	−0.211***	−0.027***	−0.193***	−0.025***
	(0.017)	(0.002)	(0.017)	(0.002)
Fairness perception			0.290***	0.036***
			(0.030)	(0.004)
Control variables	Yes	Yes	Yes	Yes
Provinces	Yes	Yes	Yes	Yes
F value/Wald test	14.379***	575.417***	15.998***	635.450***
R^2^/Pseudo R^2^	0.120	0.042	0.139	0.049
Observations	5403	5403	5106	5106

***Notes:*** ***p < 0.01. Standard errors are presented in parentheses. The contents in the table are calculated based on CSS data.

**Table A4:** Omitted variable test based on [43].

Test method	Judging criteria	Actual calculation results	Whether test was passed
(1)	$\beta^{*}$ = $\beta^{*}\left(R_{\max,\delta }\right)\in \left(0.051,0.118\right)$	0.077	Yes
(2)	δ>1	7.889	Yes

***Note:*** The contents in the table are calculated based on CSS data.

**Table A5:** Robustness checks: OLS model and Oprobit model

Variables	OLS model			Oprobit model		
	（1）	（2）	（3）	（4）	（5）	（6）
Panel A: life satisfaction as the dependent variable (CSS2017)						
Food safety perception	0.272***	0.152***	0.146***	0.027***	0.015***	0.015***
	(0.033)	(0.032)	(0.032)	(0.003)	(0.003)	(0.003)
Control variables	No	Yes	Yes	No	Yes	Yes
Provinces	No	No	Yes	No	No	Yes
F value/Wald test	69.146	123.582	35.587	73.588	1060.057	1089.804
R^2^/Pseudo R^2^	0.012	0.184	0.186	0.003	0.048	0.049
Observations	6271	6271	6271	6271	6271	6271
Panel B: happiness as the dependent variable (CSS2021)						
Food safety perception	0.159***	0.103***	0.096***	0.108***	0.069***	0.063***
	(0.017)	(0.018)	(0.018)	(0.011)	(0.011)	(0.011)
Control variables	No	Yes	Yes	No	Yes	Yes
Provinces	No	No	Yes	No	No	Yes
F value/Wald test	87.278	30.560	12.208	94.115	320.924	419.289
R^2^/Pseudo R^2^	0.029	0.125	0.154	0.016	0.070	0.087
Observations	2946	2946	2946	2946	2946	2946
Panel C: life satisfaction as the dependent variable (CSS2013)						
Food safety perception	0.159***	0.099***	0.097***	0.013***	0.008***	0.008***
	(0.018)	(0.018)	(0.018)	(0.002)	(0.002)	(0.002)
Control variables	No	Yes	Yes	No	Yes	Yes
Provinces	No	No	Yes	No	No	Yes
F value/Wald test	78.943	88.646	30.951	78.279	846.311	995.484
R^2^/Pseudo R^2^	0.013	0.148	0.166	0.004	0.051	0.057
Observations	6271	6271	6271	6271	6271	6271

***Notes:*** ***p < 0.01. Standard errors are presented in parentheses. The contents in the table are calculated based on CSS data.

**Table A6:** Robustness checks: IPW method and IPWRA method estimates

Data sources	Dependent variable	Estimation method	ATT	*t* value
CSS2017	Life satisfaction	IPW	0.148 (0.056)***	2.62
		IPWRA	0.150 (0.055)***	2.72
CSS2021	Happiness	IPW	0.137 (0.032)***	4.28
		IPWRA	0.146 (0.037)***	3.95
CSS2013	Life satisfaction	IPW	0.145 (0.031)***	4.76
		IPWRA	0.134 (0.030)***	4.44

***Notes:*** ***p < 0.01. Standard errors are presented in parentheses. The contents in the table are calculated based on CSS data.

## Footnotes

2 Data source, https://xueqiu.com/2027572105/241285278.

3 CSS Database URL: http://csqr.cass.cn/.

4 The Institute of Sociology of the Chinese Academy of Social Sciences is a national level academic research institution in Chinese sociology and the largest sociology research institute in China. The URL: http://sociology.cssn.cn/swz/.

5 Like the RBOP method, the CMP approach addresses the selection bias arising from both observed and unobserved heterogeneities but they use different estimators in calculations.
